# Effects of Maxillary Movements on Lips Following Orthognathic Surgery: A Retrospective Non-Randomized Clinical Trial

**DOI:** 10.1055/s-0043-1773759

**Published:** 2024-02-05

**Authors:** Soodeh Tahmasbi, Kasra Rahimipour, Mahshid Namdari, Reza Tabrizi, Fatemesadat Seyedzadeghomi

**Affiliations:** 1 Department of Orthodontics, School of Dentistry, Shahid Beheshti University of Medical Sciences, Tehran, Iran; 2 Department of Oral and Maxillofacial Radiology, Faculty of Dentistry, Tabriz University of Medical Sciences, Tabriz, Iran; 3 Department of Community Oral Health, Faculty of Dentistry, Tehran University of Medical Sciences, Tehran, Iran; 4 Department of Oral and Maxillofacial Surgery, School of Dentistry, Shahid Beheshti University of Medical Sciences, Tehran, Iran

**Keywords:** Le Fort I osteotomy, maxillary advancement, maxillary impaction, upper lip

## Abstract

**Introduction** Lips play a fundamental role in facial attractiveness and in decisions pertaining to orthognathic surgery.

**Objective** To assess the upper lip changes following Le Fort I osteotomy for maxillary advancement and/or impaction.

**Methods** In the present retrospective non-randomized clinical trial, we evaluated 3 groups of patients who underwent Le Fort I osteotomy of the maxilla. Group 1 (n = 35) underwent maxillary advancement, group 2 (n = 14), maxillary impaction, and group 3 (n = 11) was submitted to both maxillary advancement and impaction. The lip thickness of all patients was measured preoperatively, and the participants in each group were categorized into two subgroups: thin (< 12 mm) and thick (> 12 mm) lip. The primary (before orthognathic surgery) and final (after orthodontic bracket removal) lateral cephalograms of the patients were analyzed using the Dolphin software. Comparisons were made using the paired *t* -test and linear regression in the IBM SPSS Statistics for Windows software.

**Results** The length of the upper lip increased by 1 mm ( *p* = 0.012) on average following maxillary advancement, and it decreased by 0.43 mm ( *p* = 0.24) on average following maxillary impaction. In the maxillary advancement group, the change in angulation of the incisors predicted the incisal display ( *p* = 0.03). In the maxillary impaction group, skeletal changes in the vertical dimension predicted changes in upper lip length ( *p* = 0.033).

**Conclusions** Le Fort I osteotomy for maxillary advancement significantly increases the length of the upper lip. The assessment of lip thickness prior to surgery can help predict the postoperative results. Changing the angulation of the incisors can predict the incisal display. In maxillary impaction, skeletal changes in the vertical dimension can predict the changes in the length of the upper lip.

## Introduction

Facial esthetics has a substantial impact on social and psychological wellbeing. 1 2 Some patients may require orthognathic surgery for correction of their dental malocclusion. In such cases, orthognathic surgery may affect facial appearance due to alterations in skeletal facial structures and changes in soft tissue position. 3 4 5

Advances in surgical techniques, imaging modalities, and rigid internal fixation methods have led to a short hospital stay and minimized the risks of orthognathic surgical procedures; consequently, they have greatly contributed to the increasing popularity of orthognathic surgery. As a result, currently, orthognathic surgical procedures are more commonly performed for the treatment of severe congenital or acquired deformities. 6 Eslami et al. 7 have recommended that patient's class-III malocclusion and a Wits appraisal shorter than −5.8 mm must be treated by surgery.

Orthognathic surgery is performed to correct the skeletal relationship and improve the facial appearance, which highlights the significance of a precise assessment of the effects of surgery on soft tissue parameters and ratios, and their consequent impact on facial attractiveness. Correction of malocclusion is associated with some changes in soft tissues and particularly the lips. Evidence shows alterations of the soft tissue of the upper lip following orthognathic surgery of the maxilla. 8 9 10 11 A cephalometric analysis by Ribeiro et al. 8 revealed changes in the soft tissue of the upper lip following maxillary advancement in 70% to 80% of the patients, while no changes were detected in the lower lip. Betts et al. 9 demonstrated that Le Fort I osteotomy alone or in combination with mandibular surgery resulted in a wider and longer philtral column of the upper lip.

The reported soft tissue changes following orthognathic surgical procedures have been variable. According to a systematic review, 12 changes in the upper lip length range from a reduction of 0.8 mm to an increase of 2.48 mm following Le Fort I osteotomy of the maxilla. The authors added that accurate comparison of the study results would be difficult due to variations in the type and technique of surgery, and no consensus has been reached regarding the magnitude of soft tissue changes following surgical hard tissue alterations. 12

Üstün et al. 10 reported a significant increase in upper lip length after orthognathic surgery, but they did not discuss the magnitude of this increase relative to the magnitude of the skeletal changes. Ribeiro et al. 8 reported upper lip changes only in the horizontal dimension following skeletal changes. Moreover, orthognathic maxillary surgeries have not been evaluated individually regarding soft tissue alterations, and there seems to be a gap of information regarding upper lip changes following Le Fort I osteotomy in the * race.

(In order to blind the manuscript, “*” is used in this file. The missing information is provided in the Cover Letter of this submission.).

These questions are also posed in three-dimensional studies; Jung et al. 13 observed a wide variety of soft tissue responses to the bony movement, which could be due to the process of adaptation of the soft tissue after surgery, so a study with a large sample is required to increase reliability and confirm the factors that influence the nasolabial soft tissue changes after orthognathic surgery.

Therefore, the purpose of the present study was to assess upper lip changes following Le Fort I osteotomy for maxillary impaction and advancement (individually and in combination) by comparing the pre- and postoperative lateral cephalograms of the patients.

## Materials and Methods

This retrospective non-randomized clinical trial was conducted at the Orthodontics Department of School of Dentistry, Shahid Beheshti University of Medical Sciences and a private orthodontic office from 2001 to 2018. The study was conducted in accordance with the World Medical Association Declaration of Helsinki (of 1975 as revised in 2000) and was approved by the ethics committee of Shahid Beheshti University of Medical Sciences, Tehran, Iran. (Institutional Review Board: IR.SBMU.RIDS.REC.1396.575).

### Trial Design

A retrospective non-randomized clinical trial was conducted with all patients who underwent orthodontic treatment plus Le Fort I osteotomy for maxillary advancement and/or impaction in the Orthodontics Department of the School of Dentistry, ** University of Medical Sciences, and a private orthodontic practice from 2001 to 2018. The results were reported in accordance with the guidelines of the Consolidated Standards of Reporting Trials (CONSORT).

### Participants, Eligibility Criteria, and Settings

The inclusion criteria were patients who underwent orthodontic treatment plus Le Fort I osteotomy for maxillary advancement and/or impaction with pre- and postoperative (after six months) lateral cephalograms available. The patients received thorough instructions regarding their body posture, head and neck position, and position of the teeth (relaxed or in occlusion) and lips (closed or relaxed) during radiography. The radiographs were taken in natural head position. A ruler had to be present on the photographs, and the soft tissue had to be visible.

The exclusion criteria were systemic diseases, history of trauma, lip augmentation, cleft lip and palate, facial nerve disturbances, and incomplete patient records (unavailability of photographs or pre- and/or postoperative lateral cephalograms).

**Fig. 1:**
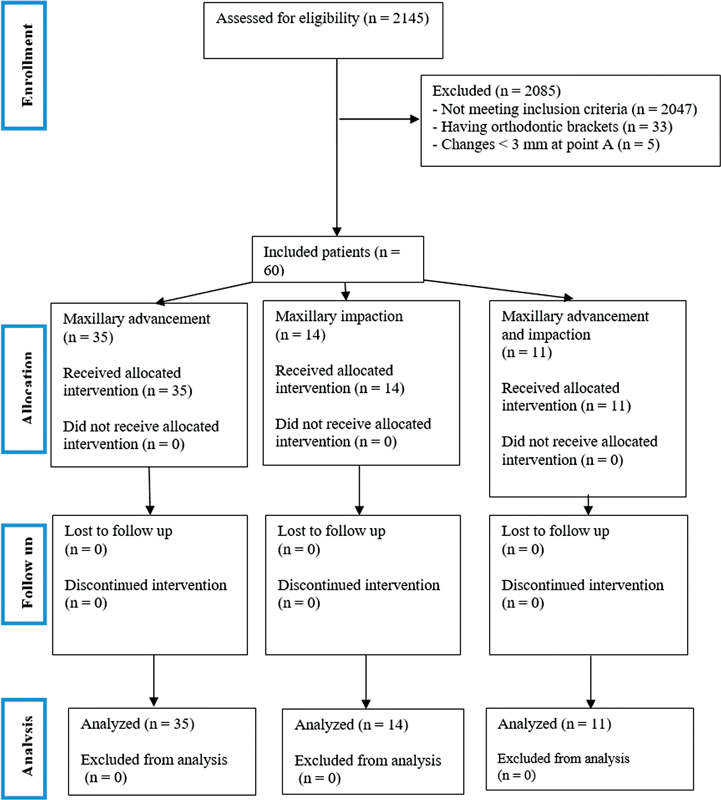
Flow diagram of the present study.

For patient selection, records of the aforementioned patients were retrieved, and 2,145 records were initially identified. After applying the eligibility criteria, a total of 98 patients remained, and their pre- and postoperative (after 6 months) lateral cephalograms were scanned (the landmarks used and the method will be described further in this section) with a laser scanner (Hewlett-Packard, Palo Alto, CA, United States) and analyzed using the Dolphin software (Dolphin Imaging and Management Solutions,, Canoga Park, CA, United States), version 10.5. After another assessment, 33 patients were excluded because they had orthodontic brackets, which can alter lip parameters. 14 We also excluded another 5 patients due to changes < 3 mm at point A in the vertical or horizontal dimensions ( Fig. 1 ). 8

### Interventions

**Fig. 2:**
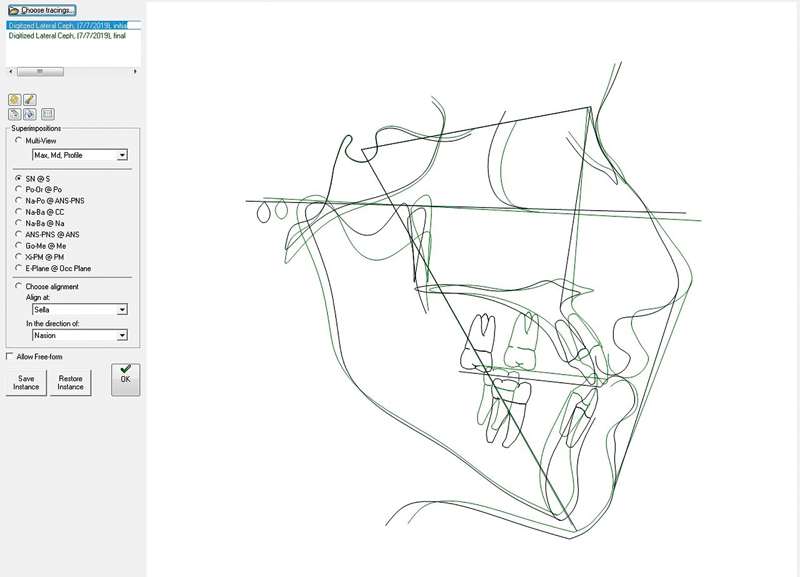
Superimposition of lateral cephalogram tracings in the Dolphin software.

Digital and manual tracings were performed to accurately measure the changes in the maxilla in the sagittal and vertical dimensions. First, the pre- and postoperative tracings were superimposed in the software to determine the hard tissue changes at point A in the vertical and horizontal dimensions ( Fig. 2 ). Next, each tracing was printed. On the printed cephalometric tracings, a horizontal line was drawn 7° below the sella-nasion (SN) line, 13 and another line was drawn perpendicular to it to manually determine the horizontal and vertical changes at point A.

To divide the patients into groups, changes > 3 mm in the horizontal dimension and < 3 mm in the vertical dimension were considered for the advancement group, while changes < 3 mm in the horizontal dimension and > 3 mm in the vertical dimension were considered for the impaction group. Cases of changes > 3 mm in both dimensions were assigned to the maxillary advancement + impaction group. 8

We have tried our best to select patients who underwent orthognathic surgery with the standard technique, so that the osteotomy incision line started from the pterygomaxillary region and extended to the pyriform aperture a few millimeters higher than the apices of the maxillary teeth. The V-Y closure (cinch suture) had been performed for all patients, and 4 plates at the zygomatic buttress and the pyriform aperture (2 per each side), as well as 16 titanium screws had been used for fixation of the bone segments.

To assess the reliability of the measurements, ten cephalograms were traced again by the same operator after ten days, and the intraclass correlation coefficient (ICC) was calculated.

### Cephalometric Analysis

The analysis performed in the Dolphin software included the following parameters: SNA, SNB, ANB, upper 1 (U1) to SN, IMPA, inter-incisal angle, upper lip length, upper lip thickness, vermilion border, maxillary incisal display, nasolabial angle, upper lip to E-plane, lower lip to E-plane, Y-axis, and the Jarabak index.

Next, all pre- and postoperative lateral cephalograms were analyzed by the same operator, and by their superimposition, the changes in hard and soft tissues were determined ( Fig. 1 ). Moreover, each group was divided into 2 subgroups according to the upper lip thickness. Patients with a lip thickness between 7.7 mm and 11.9 mm were categorized as thin, and those with lip thickness ranging from 12 mm to 19.1 mm were categorized as thick. 15 16

### Outcomes (Primary and Secondary)

The main objective of the present study was to assess the upper lip changes following Le Fort I osteotomy for maxillary impaction and advancement (individually and in combination) by comparing the pre- and postoperative lateral cephalograms of the patients.

### Sample Size Calculation

The sample size was calculated to be of 60 using the sample size estimation formula based on a two-group design, assuming an alpha of 0.05, beta of 0.2%, and study power of 80%. 14

N = [(Zα + Zβ)/C] 2 + 3;

C = 0.5 * ln[(1 + r)/(1-r)].

### Interim Analyses and Stopping Guidelines

No interim analyses were performed, and no stopping guidelines were established.

### Randomization

Randomization was not applicable to the present clinical trial.

### Blinding

The clinician who traced the cephalograms and made the measurements and the statistician who analyzed the data were blinded to the group allocation of the patients.

### Statistical Analysis

The normal distribution of the data was evaluated using the Shapiro-Wilk and Kolmogorov-Smirnov tests. The mean and standard deviation values for the upper lip length, upper lip thickness, and nasolabial angle were compared postoperatively with the baseline values using the paired *t* -test. Linear regression was applied to assess the effect of surgery (skeletal changes at point A), orthodontic changes (U1 to SN), and the effect of soft tissue thickness on changes in the length and thickness of the upper lip, the nasolabial angle, and the incisal display. All the aforementioned analyses were performed separately for each group. Linear regression and correlation tests were used to assess the correlation regarding the changes at point A in the horizontal and vertical dimensions with the upper lip thickness, nasolabial angle, and incisal display. The ICC was calculated to assess the intraexaminer reliability and the agreement between the manual and digital measurements. All statistical analyses were performed using The IBM SPSS Statistics for Windows (IBM Corp., ‘Armonk, NY, United States) software, version 23.0.

## Results

### Participant Flow

We evaluated 60 patients: 35 patients in the maxillary advancement group (21 females and 14 males), 14 patients in the maxillary impaction group (12 females and 2 males), and 11 patients in the maxillary advancement + impaction group (8 females and 3 males) with a mean age of 23.71 ± 4.95 (range: 18 to 38) years).

A total of 23 patients had only undergone maxillary surgery, 5 patients had undergone maxillary surgery plus genioplasty, and 37 patients had undergone bimaxillary surgery, 13 of whom had undergone genioplasty as well.

### Group Analyses

The Shapiro-Wilk and Kolmogorov-Smirnov tests confirmed the normal distribution of the data in all three groups ( *p* > 0.05); thus, parametric tests were applied to analyze the changes. On average, the ICC for the measurements was of 97% for all variables, which indicated excellent reliability.

The comparison of the manual and digital analyses for the assessment of the vertical and horizontal changes at point A revealed equal values in the horizontal dimension in 72%, and in the vertical dimension in 66% of the cases. The changes according to the manual analysis were as follows:

The paired *t* -test revealed that the postoperative changes in the upper lip length ( *p* = 0.043) and incisal display ( *p* = 0.018) were significant compared with the baseline values for all 60 patients. The mean amount of maxillary advancement in the advancement group was of 4.54 ± 1.71 mm at point A. The mean vertical change at point A was of 1.51 ± 0.45 mm. The upper lip length increased by 2.23 ± 1 mm in the maxillary advancement group, which was significant compared with the baseline preoperative value ( *p* = 0.012). In this group, the upper lip thickness and the nasolabial angle decreased on average by 2.03 ± 0.22 mm and 12.47 ± 0.32° respectively; these changes were not statistically significant ( *p* > 0.05; Table 1 ). The incisal display decreased by 2.03 ± 0.11 mm, which was not significant either ( *p* > 0.05; Table 1 ).

**Table 1:** Changes in upper lip parameters in the maxillary advancement group

Parameter	Mean	Standard deviation	Minimum	Maximum	*p* -value
Change in upper lip length	1.00	± 2.23	−3.5	4.8	0.012
Change in upper lip thickness	−0.22	± 2.03	−4.8	3	0.515
Change in nasolabial angle	−0.32	± 12.47	−33.8	25.1	0.877
Change in incisal display	−0.11	± 2.03	−4.5	3.3	0.747

The magnitude of the impaction of the maxilla at point A was of 4.21 ± 1.06 mm (range: 3 mm to 6 mm) in the impaction group; the mean horizontal change of the maxilla at point A was of 1.51 ± 1.14 mm in this group. The mean length and thickness of the upper lip and the size of the nasolabial angle decreased in the maxillary impaction group; however, the changes in these parameters were not statistically significant ( *p* > 0.05; Table 2 ). The incisal display significantly decreased in this group, by 1.54 ± 0.88 mm ( *p* = 0.05).

**Table 2:** Changes in upper lip parameters in the maxillary impaction group

Parameter	Mean	Standard deviation	Minimum	Maximum	*p* -value
Change in upper lip length	−0.43	± 1.32	−2.8	1.6	0.240
Change in upper lip thickness	−0.07	± 1.06	−1.8	2.3	0.787
Change in nasolabial angle	−3	± 7.54	−15.4	10.4	0.160
Change in incisal display	−0.88	± 1.54	−4	1.8	0.052

In the maxillary advancement + impaction group, the mean magnitude of the maxillary advancement at point A was of 5.13 ± 2.05 mm (range: 3 mm to 9.5 mm) while the mean magnitude of the maxillary impaction at point A was of 4.81 ± 1.67 mm (range: 3 mm to 8 mm). The upper lip length increased by 1.64 ± 0.30 mm, and the mean upper lip thickness and the size of nasolabial angle averagely decreased in this group; however, these changes were not significant ( *p* > 0.05; Table 3 ). The incisal display significantly decreased by 1.85 ± 1.57 mm ( *p* = 0.03).

**Table 3:** Changes in upper lip parameters in the maxillary advancement + impaction group

Parameter	Mean	Standard deviation	Minimum	Maximum	*p* -value
Change in upper lip length	−0.30	± 1.64	−2.2	2.6	0.543
Change in upper lip thickness	−1	± 1.71	−2.6	2.1	0.285
Change in nasolabial angle	−0.61	± 8.91	−15.7	13.5	0.824
Change in incisal display	−1.85	± 1.57	−4.3	0.4	0.03

In the maxillary advancement group, after controlling for the effect of the surgery on the horizontal skeletal changes, the changes in the angulation of incisors, the baseline lip thickness and the baseline value of the upper lip length and nasolabial angle, the results revealed that only the baseline values could predict changes. However, for the incisal display, in addition to the baseline value, the effect of the change in the U1-to-SN angle was also significant ( *p* = 0.03). In other words, the change in angulation of the incisors predicted the postoperative incisal display, and per each 1° of change in the angulation, the incisal display decreased by 0.314 mm.

In the maxillary impaction group, the relationship involving the skeletal changes and angulation of the incisors with the upper lip thickness, nasolabial angle or incisal display was not significant, but skeletal changes in the vertical dimension predicted the changes in the upper lip length, and this relationship was statistically significant ( *p* = 0.033). In other words, each 1 mm of impaction at point A decreased the upper lip length by 0.68 mm. The Pearson correlation test was applied to assess the relationship between skeletal and upper lip changes independently in each group, which revealed no significant correlation with any variable.

In the maxillary advancement group, in patients with thin lip (n = 13) the horizontal changes at point A were significantly correlated with the changes in the upper lip thickness (r = −0.663; *p* = 0.014), so, for each 1 mm of advancement at point A, the upper lip thickness decreased by 0.66 mm. There was a significant correlation between the horizontal changes at point A and the incisal display (r = −0.656; *P* = 0.015), so, for each 1 mm of advancement at point A the incisal display decreased by 0.656 mm. Moreover, the changes in the incisal display were significantly correlated with those in the angulation of the incisors (r =- 0.671; *p* = 0.012), and each 1° of increase in the angulation of the incisors decreased the incisal display by 0.671 mm.

In patients with thick lip (n = 22), a significant correlation was noted between the upper lip length and thickness postoperatively compared with the baseline values ( *p* = 0.010 and *p* = 0.024 respectively), and the upper lip length increased on average by 1.25 mm, while the upper lip thickness decreased by 1.14 mm.

In patients with thick lip in the maxillary impaction group, only the change in incisal display (decrease of 1.28 mm; *p* = 0.03) was significant postoperatively.

In patients with thick lip in the maxillary advancement + impaction group, a significant correlation was noted between the changes in the upper lip thickness and the nasolabial angle (r = −0.858; *p* = 0.029), so, for each 1° of increase in the size of the nasolabial angle, the upper lip thickness decreased by 0.85 mm.

Finally, the regression of each index was performed for all 60 patients. Regarding upper lip length, upper lip thickness, and nasolabial angle, after controlling for the aforementioned parameters, the results showed that only the baseline values predicted the changes. However, regarding the incisal display, not only the baseline values but also the change in the angulation of the incisors predicted the postoperative changes (B = −0.287; *p* = 0.03).

## Discussion

In the present study, we assessed the upper lip changes following Le Fort I osteotomy for maxillary impaction and/or advancement by comparing the pre- and postoperative lateral cephalograms of the patients.

The preoperative records were obtained prior to the initiation of the orthodontic treatment, and the patients were followed-up for 6 to 12 months postoperatively. According to Stella et al., 17 the lip thickness stabilizes six months postoperatively. Thus, assessments at the 6-month follow-up can yield more reliable results. On the other hand, evidence shows that minimal changes occur in the long-term after the surgical procedure. 6 18

The assessment of soft tissue changes after orthognathic surgery requires a three-dimensional (3D) analysis because of the complexity of the soft tissue behavior and because asymmetric areas cannot be measured accurately using two-dimensional (2D) images. CBCT has been reported to provide a concurrent and accurate representation of the hard and soft tissues with low radiation and greater dimensional accuracy. Some researchers 19 20 have suggested a significant superiority of the CBCT evaluation in comparison to the traditional 2D tracing of facial landmarks. But taking the limitations of the present study into account and considering its retrospective nature, the only option available was the traditional 2D facial landmarks on lateral cephalograms. On the other hand, there are also numerous studies 14 21 that only assess 2D facial landmarks available which are still of great importance in the literature. But it seems to be obvious that future (and especially prospective) studies should focus on using the CBCT assessment, which is more reliable and more accurate. 19

In the assessment of the upper lip changes, Altug-Atac et al. 14 reported that the greater effect of the bimaxillary surgery on the upper lip was due to the closed position of the upper lip to the surgical incision site; consequently, the surgical incision scar during the wound healing period has a greater effect on the upper lip, rather on than the lower lip and the chin area. Ribeiro et al. 8 showed that the soft tissue changes in the upper lip followed the hard tissue changes in up to 80% of the cases, while the lower lip did not undergo any significant change. Thus, the present study focused on the changes in the upper lip. Moreover, Altug-Atac et al. 14 demonstrated that postoperative changes after bimaxillary surgery were completely similar to the changes that occurred after individual surgical procedures on the maxilla or mandible. Thus, the present study evaluated three groups of patients who underwent maxillary advancement and/or impaction. In a review study, Khamashta-Ledezmaa and Naini 12 stated that factors affecting the hard and soft tissue ratios, such as patient-related factors (age and sex), ethnicity, race, magnitude and direction of the surgical movement, lip thickness, and surgical technique (such as V-Y closure, alar base cinch suture, or osseous recontouring of the anterior nasal spine), should be precisely evaluated. Accordingly, the present study focused on the * race which is a mixed-race close to the Caucasian race. (In order to blind the manuscript, “*” is used in this file. The missing information is provided in the Cover Letter of this submission.). Moreover, the magnitude and direction of the movement of the maxilla were determined based on the movement of point A in the vertical and horizontal directions, and movements > 3 mm were evaluated in the three groups. The V-Y closure was performed in all patients, who were also categorized based on their lip thickness and analyzed. The horizontal and vertical changes at point A were determined by digital software and also manually, yielding similar results in 69% of the cases.

Khamashta-Ledezmaa et al. 12 demonstrated that maxillary advancement with/without impaction increased the upper lip length (sum of SN-LS and LS-STMS) by 1.55 mm on average. Betts et al. 9 evaluated 32 patients submitted to Le Fort I osteotomy and reported an increase in the upper lip length at the philtrum. They 9 reported that maxillary advancement causes an increase in the length of the upper lip (SN-STMS) of 1 mm on average. Khamashta-Ledezmaa et al. 12 performed V-Y closure, similarly to the present study, which increases the lip vermilion display by 23%. Evidence shows that the upper lip length decreases after maxillary impaction by 30% (range: 20% to 40%) on average. Jensen et al. 22 evaluated soft tissue changes following simultaneous maxillary impaction and mandibular advancement and reported a reduction of 1.9 ± 0.8 mm in the upper lip length. In the present study, we found a reduction of 1.6 ± 0.3 mm following maxillary impaction, which was not statistically significant; this is probably due to the great variability of posttreatment lip positions. 23 However, skeletal changes in the vertical dimension significantly predicted the postoperative upper lip length changes, so, for each 1 mm impaction at point A, the upper lip length decreased by 0.68 mm.

Controversy still exists regarding the thickness of the lip. Some studies have used the Pearson correlation and showed that the upper lip thickness was influenced by its baseline preoperative thickness, while some others reported no change. 8 24 Moreover, it has been claimed that thin lips follow the hard tissue changes more than thick lips. 17

Different values have been proposed for to classify patients as having thin or thick lips. In the present study, and in line with the study by0 Khamashta-Ledezmaa et al., 12 lip thickness < 12 mm was considered as thin, and higher values were considered as thick.

Betts et al. 9 demonstrated that the upper lip thickness generally increased after maxillary osteotomy, irrespective of the type of surgery, while Stella et al. 17 reported a 2-mm reduction following maxillary advancement. The present study conformed to the results reported by Stella et al. 17 regarding the reduction of lip thickness in all three groups; however, this change was not significant without grouping the patients based on their preoperative lip thickness. In patients with thin lips, each 1 mm of advancement of the maxilla decreased the upper lip thickness by 0.66 mm according to the linear regression. In patients with thick upper lips, a reduction of 1.14 mm was noted, which was in agreement with the results reported by Ribeiro et al., 8 who showed that soft tissue at the maxillary position (the most anterior-inferior point on the alveolar ridge between the incisors) advanced by 70% per each 1 mm o advancement, which indicates that the upper lip thickness at the vermilion decreased after surgery by 30%, or 0.3 mm. In agreement with the aforementioned findings, Altug-Atac et al. 14 divided the upper lip soft tissue into two segments and measured the surface area of each segment. They reported a reduction in both segments. Bays et al., 25 reported thinning of the upper lip as the result of anterior displacement of the maxilla, and Möhlhenrich et al. 26 also reported upper lip thickness maxillary advancement versus mandibular setback or bimaxillary surgery (A–A’ on NL) decrease significantly.

Chalipa et al. 27 and Betts et al. 9 evaluated Le Fort I osteotomy in general and reported a reduction in the nasolabial angle postoperatively, which was also noted in all groups in the present study, and the mean reduction was greater in the maxillary impaction group; however, it was not significant in any group. After grouping the patients based on their upper lip thickness, however, a significant correlation was noted between the changes in the upper lip thickness and the changes in the nasolabial angle in patients with thick upper lip who underwent maxillary advancement + impaction, so, for each 1° of increase in the size of the nasolabial angle, the upper lip thickness decreased by 0.85 mm.

Shmuly et al. 28 evaluated 32 patients who underwent maxillary advancement and reported a reduction of 9.64 ± 3.78° in the nasolabial angle, but found no correlation between the skeletal and soft tissue changes. In a prospective study, Khamashta-Ledezmaa and Naini 29 reported an average increase in the nasolabial angle of 1.88°; the main reason was the upward movement of the nasal columella, and the share of the advancement of the maxilla and cinch suture was 52% in this outcome.

Orthognathic surgery to improve facial esthetics requires comprehensive knowledge about the correct ratios and skeletal and soft tissue changes. 21 Ribeiro et al. 8 demonstrated a strong correlation between hard and soft tissue point A in the horizontal analysis, so each 1% change in AH caused a 0.859% change in the AS point. However, there are discussions in the literature claiming that there is a space between the incisors and the upper lip, which is primarily decreased by maxillary advancement before impacting the upper lip. 8 17 In the study by Khamashta-Ledezmaa and Naini, 29 the incisal display at rest increased by 0.5 mm on average, while advancements of 3.4 mm impactions of 1.4 mm impaction on average were performed. They 29 stated that soft tissue manipulation and V-Y and AC sutures could affect soft tissue response and incisal display in Le Fort I osteotomy for maxillary advancement with/without impaction. They did not find a significant correlation between skeletal changes and incisal display when the lips were at rest.

The present study indicated that, in maxillary advancement, changing the angulation of incisors could predict the postoperative incisal display, and each 1° of change in angulation of incisors decreased the incisal display by 0.314 mm. Moreover, a significant correlation was noted between the horizontal changes at point A and the incisal display in patients with thin lip. The incisal display decreased in the maxillary impaction and maxillary advancement + impaction groups.

### Limitations

The present study had some limitations. Due to its retrospective design, we could not standardize the patients regarding certain factors. Moreover, the patients had been operated on by different surgeons, which can affect the outcome. The magnitude of the maxillary advancement and impaction was variable, which could have affected the results. However, by increasing the sample size, the relationship between the magnitude of the surgical skeletal changes and the upper lip changes could be studied. Similar prospective studies are required to obtain a complete set of pre- and postorthodontic and pre- and postsurgical photographs and cephalograms. Moreover, it would be ideal if one surgeon performs all the procedures. All cephalograms and photographs should be preferably obtained in the same center under completely standardized conditions. Moreover, further studies with larger samples are required to employ 3D analyses such as 3D photogrammetry to obtain more accurate results.

## Conclusion

The present study shows that Le Fort I osteotomy for maxillary advancement significantly increases the upper lip length. Changing the angulation of the incisors can predict the incisal display. In Le Fort I osteotomy for maxillary impaction, skeletal changes in the vertical dimension can predict the changes in the upper lip length.
